# Case Report: Prenatal diagnosis and genetic counseling for a homozygous segment spanning chromosome 6q25.3q27 with normal phenotype

**DOI:** 10.3389/fgene.2026.1935109

**Published:** 2026-10-15

**Authors:** Dandan Li, Jie Hu, Ji Wu, Xuna Bian

**Affiliations:** 1 Department of Medical Laboratory Technology, Shiyan Maternal and Child Health Hospital, Shiyan, Hubei, China; 2 College of Life Science and Technology, Huazhong University of Science and Technology, Wuhan, Hubei, China; 3 Department of Medicine, City University of Wuhan, Wuhan, Hubei, China; 4 Department of Nursing, Tongji Hospital, Tongji Medical College, Huazhong University of Science and Technology, Wuhan, Hubei, China

**Keywords:** chromosomal microarray analysis, noninvasive prenatal testing, prenatal ultrasound, runs of homozygosity, uniparental disomy, whole-exome sequencing

## Abstract

**Background:**

Runs of Homozygosity (ROH) are long, uninterrupted stretches of the genome in which an individual is homozygous at virtually every assayed variant. Uniparental disomy (UPD) is an epigenetic anomaly in which both copies, or a portion of a pair of homologous chromosomes, are inherited from one parent.

**Materials and Methods:**

A 29-year-old gravida 2, para 1 woman underwent amniocentesis at 18 weeks’ gestation because non-invasive prenatal testing (NIPT) indicated a high probability of chromosome 11 gain. GTG-banding karyotype analysis was performed on cultured amniocytes. Chromosomal microarray analysis (CMA), whole-exome sequencing (WES) on uncultured amniocytes were performed.

**Results:**

CMA detected a copy-neutral 10.42 Mb ROH of chromosome 6, arr [GRCh37] 6q25.3q27 (157,776,570_168,194,615)x2 hmz. Trio-WES confirmed that the homozygous 6q25.3q27 segment resulted from biparental inheritance. Across the 10.42 Mb interval, multiple informative SNPs showed that the fetal homozygous genotypes were compatible with the contribution of one allele from each parent, and no informative single-nucleotide polymorphism (SNP) showed fetal homozygosity with one parent being wild-type for the fetal allele, thus excluding segmental uniparental disomy. At the 6-month checkup, the baby was developing normally.

**Conclusion:**

We provide a detailed description of a homozygous segment spanning chromosome 6q25.3q27 with normal phenotype. Combination of NIPT, prenatal ultrasound, karyotype analysis, CMA, WES and genetic counseling will prove a more accurate risk assessment for the prenatal diagnosis of ROH and UPD.

## Introduction

Runs of Homozygosity (ROH) are long, uninterrupted stretches of the genome in which an individual is homozygous at virtually every assayed variant. In other words, the two homologous chromosomes carry identical alleles across this region, as if the same ancestral haplotype was inherited from both parents (Ceballos et al., 2018; Ferenčaković et al., 2013). ROH can arise from multiple causes including identity-by-descent related to parental consanguinity, population genetic drift, positive selection, as well as chromosomal events such as uniparental disomy (UPD) and mitotic recombination (Ceballos et al., 2018; Ferenčaković et al., 2013).

UPD is an epigenetic anomaly in which both copies, or part of a pair of homologous chromosomes, are inherited from one parent (Cheng et al., 2025; Yu et al., 2022). It originates from mechanisms including monosomic or trisomic rescue during early embryogenesis, chromosome segregation errors, and mitotic recombination (Cheng et al., 2025; Zeng et al., 2023). UPD is categorized into complete isodisomy, complete heterodisomy, and mixed iso-/heterodisomy. It may affect an entire chromosome or only a chromosomal segment; segmental UPD frequently derives from rescue events subsequent to chromosomal rearrangements (Liehr, 2010).

Clinically, UPD may lead to disease by disrupting imprinted gene allelic expression, revealing autosomal-recessive pathogenic variants through homozygosity, or secondary to cryptic trisomic rescue. Most ROH-related clinical phenotypes stem from homozygous pathogenic variants within the ROH interval, which unmask autosomal recessive disorders (Zhu et al., 2021; Zhu et al., 2022). Notably, UPD represents only one source of ROH; many ROH segments originate from biparental transmission of identical ancestral haplotypes and are not attributable to UPD.

Neither ROH nor UPD alters chromosome copy number or gross chromosomal architecture; therefore, conventional G-banded karyotyping cannot detect these findings (Song et al., 2021). Instead, single-nucleotide polymorphism (SNP)-based chromosomal microarray analysis (CMA), microsatellite genotyping, or trio-based whole-exome sequencing (WES) are required for identification (Song et al., 2021). Of clinical relevance for prenatal settings, chromosome 6 carries imprinted loci, meaning segmental or full-chromosome UPD involving chromosome 6 carries potential phenotypic risk that warrants careful prenatal evaluation.

Here we present a rare case of a homozygous segment spanning chromosome 6q25.3q27, with a normal post-natal clinical phenotype.

## Case presentation

A 29-year-old gravida 2, para 1 woman underwent amniocentesis at 18 weeks’ gestation because non-invasive prenatal testing (NIPT) indicated a high probability of chromosome 11 gain.

The patient and her 32-year-old husband are non-consanguineous. The family history is unremarkable for birth defects or genetic disorders.

GTG-banding karyotype analysis at 550-band resolution was performed on cultured amniocytes and parental blood samples.

CMA and trio-WES on uncultured amniocytes were performed.

CMA using the Affymetrix CytoScan 750K chip. The chip includes 550k non-polymorphic markers and 200k SNP markers, provides a high-density coverage of the genome, with probes spaced at an average interval of approximately 1.7 kb (though this may vary slightly depending on the genomic region). This density allows for detailed detection of copy number variations (CNVs) and copy-neutral loss of heterozygosity (LOH) (Xu et al., 2020).

Trio-WES and parental-origin analysis: Genomic DNA was extracted from uncultured amniocytes (fetus) and peripheral blood (both parents). WES was performed using the Nano WES capture kit (Berry Genomics, Beijing, China) on the NovaSeq 6000 platform (Illumina, San Diego, CA, USA) with a 150 bp paired-end sequencing configuration. The mean sequencing depth was approximately 100×, and 98.61% of the target region was covered at ≥20× in the fetal sample; the corresponding values for the parental samples were similar (99.51% at ≥20×). Raw image files were processed using CASAVA v1.82 for base calling. Sequencing reads were aligned to the human reference genome (hg38/GRCh38) using Burrows-Wheeler Aligner (BWA), and PCR duplicates were removed using Picard v1.57 (http://picard.sourceforge.net/). Variant calling was performed using the Verita Trekker® Variants Detection System (Berry Genomics) together with GATK (https://software.broadinstitute.org/gatk/). Variant annotation and interpretation were conducted using ANNOVAR and the Enliven® Variants Annotation Interpretation System (Berry Genomics). Variants with a minor allele frequency (MAF) < 1% in the exonic region or with a potential splicing impact were retained for deep interpretation. Interpretation considered ACMG category, evidence of pathogenicity, clinical synopsis, and the inheritance model of the associated disease. Variants were classified into five categories—pathogenic, likely pathogenic, uncertain significance, likely benign, and benign—according to the American College of Medical Genetics and Genomics (ACMG) guidelines for the interpretation of genetic variants (Tian et al., 2025).

To determine whether the 6q25.3q27 ROH arose from biparental inheritance or from UPD, we performed a trio SNP-based analysis using the WES data. The analysis was performed exome-wide across all captured regions, and the 6q25.3q27 interval was then examined in detail. Prior to SNP genotype comparison across the 6q25.3q27 interval, liftOver genome conversion was applied to translate the CMA-defined GRCh37 ROH boundaries into GRCh38 coordinates, ensuring consistent positional referencing for variant extraction from GRCh38-aligned WES BAM files. For each SNP within the analyzed regions, the fetal genotype was compared with the maternal and paternal genotypes. Parental origin was inferred using the following rules, implemented in a proprietary algorithm (Chinese Invention Patent No. ZL 202110263133.8):

If the fetus was homozygous for an allele and the father was wild-type while the mother was heterozygous or homozygous for that allele, the finding supported maternal UPD.

If the fetus was homozygous for an allele and the mother was wild-type while the father was heterozygous or homozygous for that allele, the finding supported paternal UPD.

If the fetus was homozygous and both parents were heterozygous, or one parent was homozygous and the other parent carried the same allele, the fetal homozygous genotype was compatible with biparental inheritance and did not support UPD. UPD was excluded when, across the interval, multiple informative SNPs showed that both parents contributed alleles to the fetal homozygous genotype, and no informative SNP showed fetal homozygosity with one parent being wild-type for the fetal allele. The UPD/ROH analysis was performed on an exome-wide basis rather than being restricted to 6q25.3q27; the 6q25.3q27 interval was selected for detailed presentation because it was the only region flagged by CMA as a copy-neutral ROH in the fetus.

## Results

Chromosomal GTG-banding at 550-band resolution on cultured amniocytes revealed a karyotype of 46,XX. No numerical or structural abnormalities of chromosome 11 were observed on G-banded karyotyping. Consistently, CMA did not identify copy-number alterations involving chromosome 11. Therefore, the initial NIPT finding suggestive of chromosome 11 gain was classified as a false-positive result. CMA detected a copy-neutral 10.42 Mb ROH of chromosome 6, arr [GRCh37] 6q25.3q27 (157,776,570_168,194,615)x2 hmz (Figure 1). For trio-WES-based parental-origin analysis, the GRCh37 CMA coordinates of the ROH segment were converted to GRCh38 using the UCSC Genome Browser liftOver tool to harmonize genomic positions between platforms. The equivalent GRCh38 coordinates for this ROH segment are chr6:158472728-168889404. Genome-wide CMA analysis revealed no additional copy-neutral regions of homozygosity (ROH/LOH) across the remaining autosomes. The total autosomal ROH burden for the fetus was therefore 10.42 Mb, represented exclusively by this single segment on chromosome 6.

**FIGURE 1:**
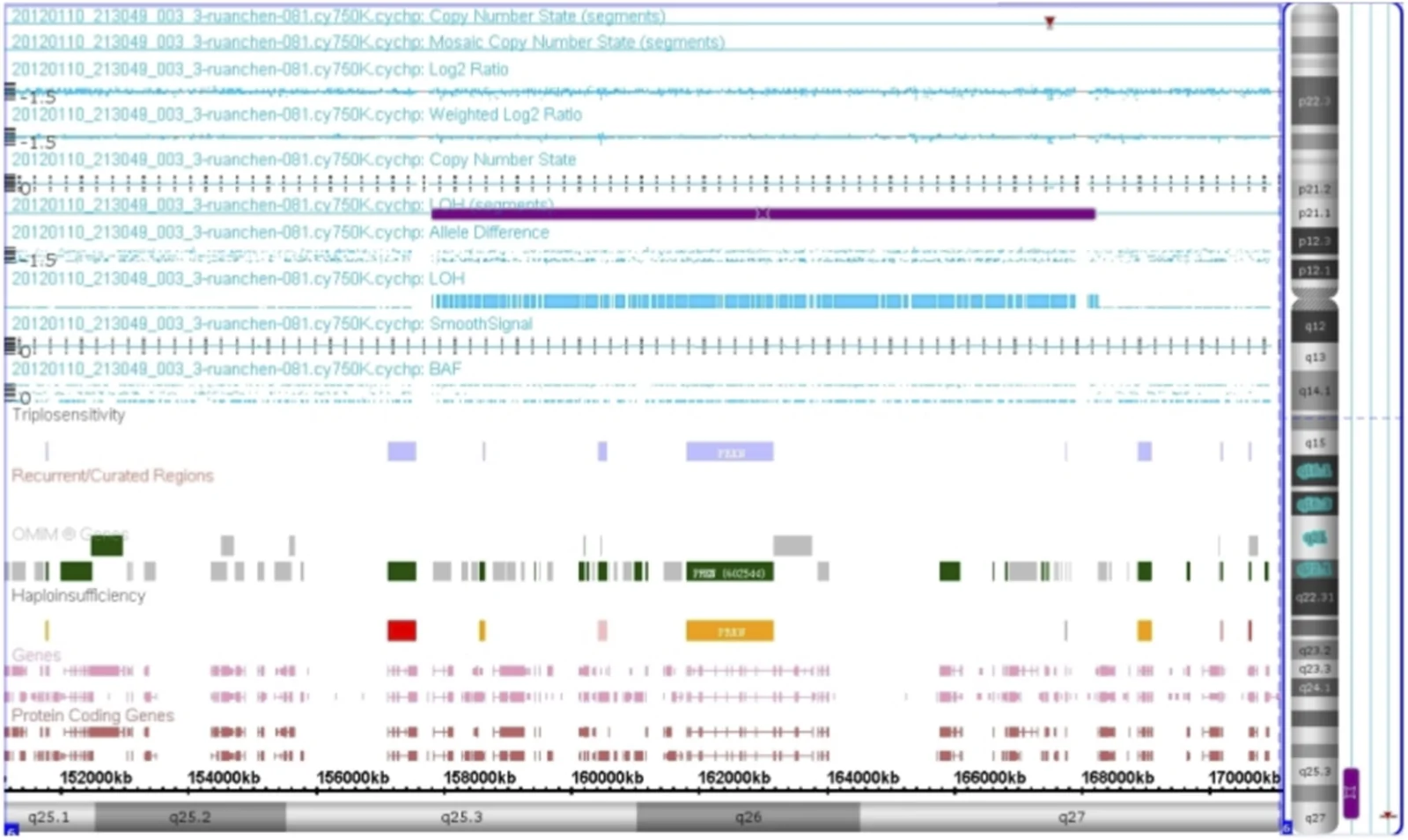
CMA detected a 10.42 Mb ROH of chromosome 6, arr [GRCh37] 6q25.3q27 (157,776,570_168,194,615)x2 hmz in the fetus.

The parental karyotypes and CMA analysis were normal.

All prenatal laboratory data were within normal range. Detailed prenatal anatomical ultrasound survey and dedicated fetal echocardiography were performed. A single umbilical artery (SUA) was identified. No additional structural malformations, facial dysmorphisms, cardiac anomalies, renal anomalies, or intrauterine growth restriction (IUGR) were detected. Therefore, this SUA was classified as isolated single umbilical artery.

After genetic counseling, we performed trio-WES on this family. No pathogenic or likely pathogenic homozygous variants associated with autosomal-recessive disease were identified by trio-WES in known recessive pathogenic genes for inherited disorders within chromosome 6q25.3q27. By analyzing SNP genotypes within the 6q25.3q27 region, trio-WES showed that the fetal homozygous genotypes were compatible with biparental inheritance: at informative SNPs, the fetus was homozygous while both parents were heterozygous, or one parent was homozygous and the other parent carried the same allele. No informative SNP showed fetal homozygosity with one parent being wild-type for the fetal allele. In the present case, the 6q25.3q27 interval contained 66 informative SNPs distributed across the entire 10.42 Mb region, all of which were compatible with biparental inheritance. These findings confirmed that the 10.42 Mb ROH was biparentally inherited and excluded segmental UPD(6). A trio SNP/haplotype figure showing fetal, maternal, and paternal B-allele frequency patterns across chromosome 6 is presented in Figure 2.

**FIGURE 2:**
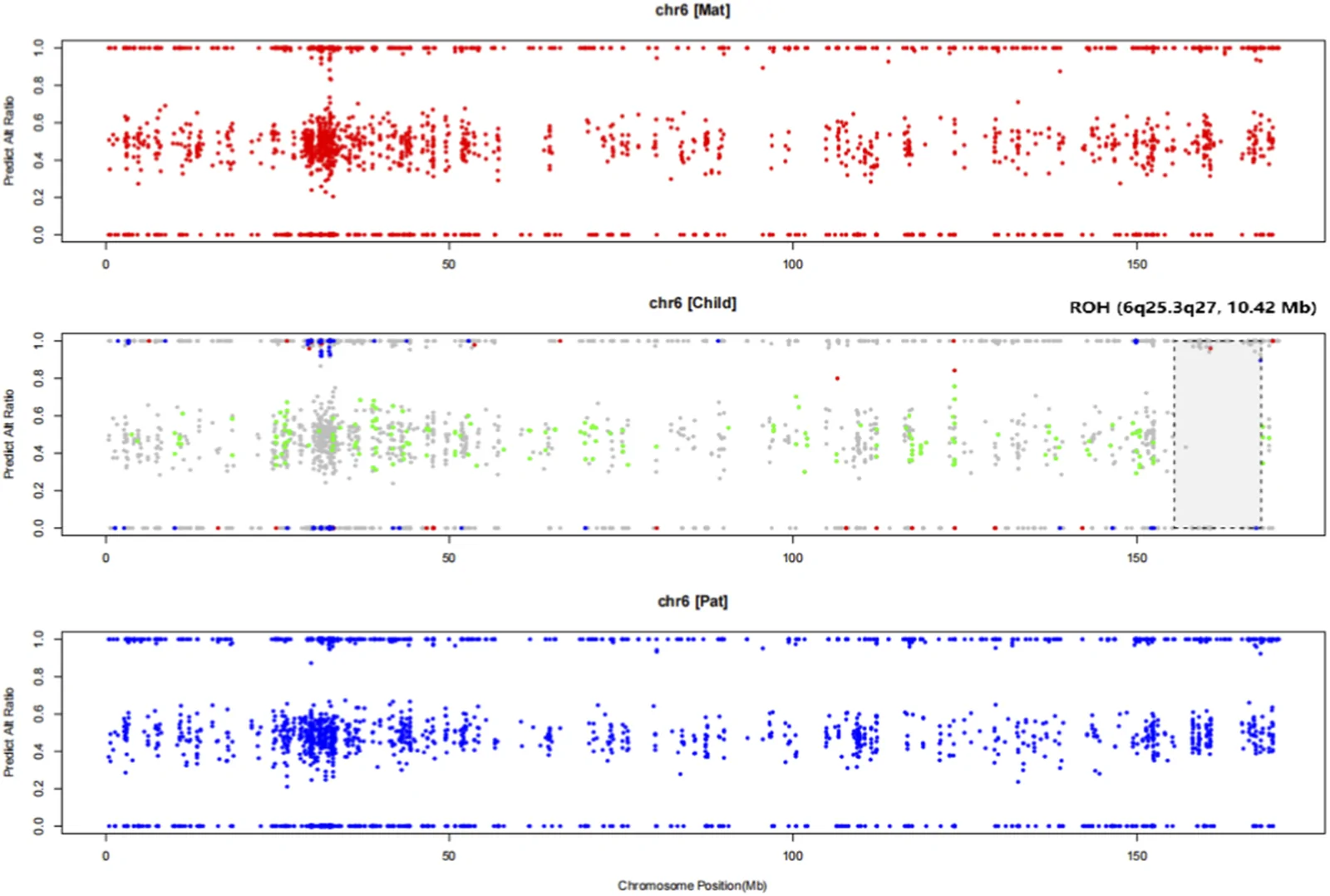
Trio-based SNP/haplotype analysis of B-allele frequency patterns across chromosome 6 in the fetus-mother-father trio. Trio-based SNP/haplotype analysis of B-allele frequency patterns across chromosome 6 in the fetus-mother-father trio. The horizontal axis denotes genomic positions on chromosome 6 (GRCh38). The boxed region highlights the 10.42 Mb biparentally-inherited ROH interval chr6:158472728-168889404 (6q25.3q27). Color legend: red, variant allele derived exclusively from the mother; blue, variant allele derived exclusively from the father; green, phased heterozygous variant with confirmed parental origin (e.g. fetus heterozygous, father heterozygous, mother wild-type, variant inherited from father); gray, other genotypic configurations (including fetus homozygous with both parents heterozygous; fetus heterozygous with both parents heterozygous, and other compatible biparental inheritance patterns). Multiple informative SNPs within the boxed ROH segment support biparental contribution and exclude segmental uniparental disomy of chromosome 6.

After genetic counseling again, the parents decided to continue the pregnancy. At 40^+2^ weeks of gestation, a 3450-g female infant was born via vaginal delivery. The 1-min, 5-min, and 10-min Apgar scores were 9, 10, and 10, respectively. The infant received a complete physical examination with normal findings. At the 6-month follow-up visit, the infant underwent a comprehensive pediatric evaluation, including anthropometric growth parameters (weight, length, head circumference), routine physical examination, and neurologic assessment performed by a pediatrician. All growth indices were within normal age-matched reference values. Motor, social-communicative, visual-auditory performances and neurological status were consistent with expected developmental milestones for age, with no clinical concerns identified.

## Discussion

NIPT is widely used in the screening of common fetal chromosome aneuploidy (Eltabbakh et al., 2024). Conventional karyotyping provides genome-wide cytogenetic assessment to detect numerical and structural chromosomal aberrations. CMA and WES serve complementary roles in prenatal diagnosis: CMA identifies copy-number variations and loss-of-heterozygosity (Xu et al., 2024), while WES detects single-nucleotide variants and small insertions-deletions (Tian et al., 2025).

In the present case, invasive prenatal work-up ruled out any chromosome 11 aberration, confirming that the initial NIPT indication of chromosome 11 gain represented a false-positive result. NIPT is a screening test rather than a diagnostic assay; false-positive NIPT findings for autosomal copy-number changes are well-documented and can originate from multiple biological sources including confined placental mosaicism, maternal copy-number variants, circulating maternal tumour DNA, or technical noise, even with adequate fetal fraction (Eltabbakh et al., 2024). This case underscores the principle that all high-risk NIPT screening results require confirmatory invasive prenatal diagnosis, rather than clinical decision-making based solely on NIPT output.

Unlike numerical or structural chromosomal anomalies, ROH and UPD do not change chromosome number or gross structure and are therefore undetectable by conventional cytogenetic karyotyping (Eltabbakh et al., 2024). SNP-based CMA can identify regions of homozygosity; however, this platform only reliably detects isodisomy and cannot identify heterodisomy (Song et al., 2021). Microsatellite analysis or trio-WES are required to discriminate among isodisomy, heterodisomy, mixed iso-/heterodisomy, and segmental UPD (Eggermann, 2020; Scuffins et al., 2021). Accordingly, any ROH identified by CMA in prenatal workup requires follow-up testing to distinguish biparentally inherited ROH from segmental UPD (Ceballos et al., 2018; Ferenčaković et al., 2013).

The major clinical risk for ROH derives from homozygous pathogenic variants within the homozygous segment, which unmask autosomal-recessive disease. By contrast, UPD may cause clinical phenotypes via three mechanisms: perturbed imprinted-gene expression, homozygosity of autosomal-recessive variants, or cryptic mosaic aneuploidy. Imprinting-related phenotypes are restricted to certain human chromosomes; chromosomes 6, 7, 11, 14, 15, and 20 harbour known imprinted loci, so UPD involving these chromosomes carries higher phenotypic risk (Del Gaudio et al., 2020). However, phenotypic consequences of chromosome-6 uniparental disomy are highly dependent on (1) parental origin (maternal versus paternal), and (2) whether the UPD affects the entire chromosome or only a chromosomal segment. For full-chromosome maternal UPD(6), clinical associations include IUGR, preterm birth, and variable neurodevelopmental phenotypes; full-chromosome paternal UPD(6) and segmental paternal UPD involving the 6q24 imprinting control region cause transient neonatal diabetes mellitus (TNDM) (Docherty et al., 2013; Eggermann et al., 2017; Jiang et al., 2024; Świeca et al., 2025). Critically, the well-characterized imprinted locus and imprinting-control region for TNDM map to 6q24, which lies proximal to our reported 6q25.3q27 ROH interval. Upon review of curated imprinted gene databases, the 10.42 Mb 6q25.3q27 segment in the present case does not overlap any established, clinically relevant imprinted genes or imprinting-control regions. Therefore, any phenotypic risk from segmental UPD limited exclusively to 6q25.3q27 would theoretically arise predominantly from unmasking autosomal-recessive pathogenic variants rather than from disrupted genomic imprinting. Even so, distinguishing segmental UPD from biparentally inherited ROH remains clinically prudent for prenatal counselling, given the general precedent for chromosome-6 UPD phenotypes reported in the literature (Cheng et al., 2025; Jiang et al., 2024).

The genome-wide ROH profile in this fetus showed only one discrete 10.42 Mb ROH on chromosome 6 without additional autosomal ROH segments. This pattern is inconsistent with the multiple, widely distributed ROH segments typically observed in offspring of consanguineous couples or remotely related parents, in which total autosomal autozygosity is substantially elevated. Instead, the observation of a single isolated ROH segment is most parsimoniously explained by transmission of a shared ancestral haplotype present in the general population inherited independently from each parent, consistent with population-level shared ancestry rather than cryptic parental relatedness.

In our prenatal case, CMA detected a 10.42 Mb ROH spanning 6q25.3q27. This genomic interval contains multiple clinically relevant genes, including *ARID1B, WTAP, DLL1, TBP, SYNJ2, TIAM2, PHF10, MAP3K4,* and *PSMB1* (Herrada-Pineda et al., 2025; Jiang et al., 2024; Li et al., 2021; Tolezano et al., 2024). Notably, none of these genes within 6q25.3q27 are well-documented imprinted loci. Pathogenic variants in several of these genes are linked to developmental disorders. In this fetus, trio-WES confirmed biparental inheritance of the identical haplotypes across this region. Specifically, across the 10.42 Mb interval, 66 informative SNPs were identified; at these sites, the fetal homozygous genotypes were consistently explained by the transmission of one allele from each parent, and no site showed fetal homozygosity with one parent being wild-type for the fetal allele. This pattern formally excluded segmental UPD(6) and supported a biparentally inherited ROH. No homozygous recessive pathogenic variants were identified within the 6q25.3q27 interval. Prenatal ultrasound identified an isolated single umbilical artery; no other major structural anomalies including facial dysmorphism or intrauterine growth restriction were detected.

Even after excluding UPD and identifiable homozygous pathogenic variants by CMA and trio-WES, important residual genetic risks remain for this prenatal case. First, whole-exome sequencing primarily targets coding exons and splice-site boundaries; it cannot reliably detect deep-intronic variants, distant regulatory-element variants, repeat expansions, certain complex structural variants, or low-level somatic mosaicism. Second, WES does not assess DNA methylation status, so epigenetic abnormalities including imprinting defects cannot be fully ruled out by short-read exome sequencing alone. Third, an isolated single umbilical artery was noted prenatally. Although isolated SUA is most often a benign incidental finding, it carries weak statistical association with subtle developmental risks that cannot be entirely excluded prenatally. In this case, there is no compelling molecular evidence supporting a causal link between this biparentally-inherited 10.42 Mb ROH and SUA. Collectively, these residual risks should be explicitly communicated to families during prenatal genetic counselling to achieve balanced risk assessment.

For prenatal counselling when encountering 6q ROH: (1) it is critical to differentiate biparental ROH from segmental UPD given the imprinting potential of chromosome 6; (2) integration of multiple lines of evidence—NIPT results, detailed prenatal ultrasound, karyotype, CMA, and trio-WES—rather than a single molecular test is essential for risk stratification; (3) long-term postnatal clinical follow-up is recommended even when no other major prenatal structural anomalies are identified besides isolated incidental ultrasound findings.

Published prenatal cases involving chromosome-6-related ROH and UPD are summarized in Table 1 for comparison. In previously reported prenatal UPD(6) cases, intrauterine growth restriction was frequently observed for maternal UPD(6) (Eggermann et al., 2017; Jiang et al., 2024; Świeca et al., 2025), whereas cases with biparental ROH of 6q without identifiable pathogenic variants may have normal postnatal outcomes (Jiang et al., 2024). Our present case adds to this limited literature: a large 10.42 Mb biparentally-derived 6q25.3q27 ROH with normal infant development at six-month follow-up.

**TABLE 1:** Comparison of the present case with published chromosome 6 ROH/UPD cases.

References	Genomic region/type	Parental origin	Prenatal findings	Molecular testing	Pregnancy outcome	Postnatal phenotype
Present case	6q25.3q27 ROH, 10.42 Mb	Biparental (UPD excluded)	NIPT: high risk of chromosome 11 gain; single umbilical artery; no IUGR or dysmorphism	G-banding; SNP-based CMA; trio-WES	Live birth at 40 + w, 3450 g; apgar 9/10/10	Normal physical examination; normal development at 6 months
Eggermann et al., 2017 (case 1)	6p12.3q27 segmental UPD	Maternal	IUGR	SNP-based CMA; MS-SNuPE/MLPA: hypermethylation of PLAGL1 and IGF2R DMRs	Term delivery; placental dysfunction	Facial dysmorphism; developmental and language delay
Eggermann et al., 2017 (case 2)	6p25.3q27 segmental UPD	Maternal	IUGR; oligohydramnios	SNP-based CMA; homozygous CYP21A2 exon 1–8 deletion; methylation analysis as above	Term delivery	Congenital adrenal hyperplasia; developmental delay
Jiang et al., 2024 (2 cases)	Whole chromosome 6 UPD	Maternal	IUGR (one with abdominal cyst)	CMA-seq; clinical exome sequencing; SNP genotyping	Termination of pregnancy (both)	Not applicable
Świeca et al., 2025	Whole chromosome 6 isodisomy	Maternal	IUGR; polyhydramnios	Trio-WES	Preterm delivery; SGA	Autism spectrum disorder; sacrococcygeal teratoma; developmental delay; dysmorphic features
Docherty et al., 2013 (cohort review)*	6q24 imprinted region (UPD or duplication)	Paternal	Mostly ascertained postnatally; IUGR reported in a subset	Methylation-specific MLPA; genotyping	Variable	Transient neonatal diabetes mellitus; macroglossia; macroglossia/craniofacial abnormalities in partial paternal UPD(6)

Abbreviations: MLPA, multiplex ligation-dependent probe amplification; MS-SNuPE, methylation-specific single-nucleotide primer extension; DMR, differentially methylated region.

* 6q24 transient neonatal diabetes mellitus cohort; partial paternal UPD of distal 6q has been reported in a single infant with neonatal diabetes, macroglossia and craniofacial abnormalities (cited in Eggermann, 2020).

## Conclusion

To summarize, we provide a detailed description of a homozygous segment spanning chromosome 6q25.3q27 with normal phenotype. Combination of NIPT, prenatal ultrasound, karyotype analysis, CMA, WES and genetic counseling will prove a more accurate risk assessment for the prenatal diagnosis of ROH and UPD.

## Data Availability

The original contributions presented in the study are included in the article/supplementary material, further inquiries can be directed to the corresponding author.
